# The influence of push-off timing in a robotic ankle-foot prosthesis on the energetics and mechanics of walking

**DOI:** 10.1186/s12984-015-0014-8

**Published:** 2015-02-22

**Authors:** Philippe Malcolm, Roberto E Quesada, Joshua M Caputo, Steven H Collins

**Affiliations:** Department of Movement and Sports Sciences, Ghent University, Ghent, Belgium; Department of Mechanical Engineering, Carnegie Mellon University, Pittsburgh, PA USA; Robotics Institute, Carnegie Mellon University, Pittsburgh, PA USA

**Keywords:** Biomechatronics, Ankle, Prosthesis, Walking, Metabolic, Timing, Push-off, Collision, Gait, Bionics

## Abstract

**Background:**

Robotic ankle-foot prostheses that provide net positive push-off work can reduce the metabolic rate of walking for individuals with amputation, but benefits might be sensitive to push-off timing. Simple walking models suggest that preemptive push-off reduces center-of-mass work, possibly reducing metabolic rate. Studies with bilateral exoskeletons have found that push-off beginning before leading leg contact minimizes metabolic rate, but timing was not varied independently from push-off work, and the effects of push-off timing on biomechanics were not measured. Most lower-limb amputations are unilateral, which could also affect optimal timing. The goal of this study was to vary the timing of positive prosthesis push-off work in isolation and measure the effects on energetics, mechanics and muscle activity.

**Methods:**

We tested 10 able-bodied participants walking on a treadmill at 1.25 m · s^−1^. Participants wore a tethered ankle-foot prosthesis emulator on one leg using a rigid boot adapter. We programmed the prosthesis to apply torque bursts that began between 46% and 56% of stride in different conditions. We iteratively adjusted torque magnitude to maintain constant net positive push-off work.

**Results:**

When push-off began at or after leading leg contact, metabolic rate was about 10% lower than in a condition with Spring-like prosthesis behavior. When push-off began before leading leg contact, metabolic rate was not different from the Spring-like condition. Early push-off led to increased prosthesis-side *vastus medialis* and *biceps femoris* activity during push-off and increased variability in step length and prosthesis loading during push-off. Prosthesis push-off timing had no influence on intact-side leg center-of-mass collision work.

**Conclusions:**

Prosthesis push-off timing, isolated from push-off work, strongly affected metabolic rate, with optimal timing at or after intact-side heel contact. Increased thigh muscle activation and increased human variability appear to have caused the lack of reduction in metabolic rate when push-off was provided too early. Optimal timing with respect to opposite heel contact was not different from normal walking, but the trends in metabolic rate and center-of-mass mechanics were not consistent with simple model predictions. Optimal push-off timing should also be characterized for individuals with amputation, since meaningful benefits might be realized with improved timing.

**Electronic supplementary material:**

The online version of this article (doi:10.1186/s12984-015-0014-8) contains supplementary material, which is available to authorized users.

## Background

Ankle-foot prostheses have gone through an impressive evolution over the last half century. In some areas of human performance such as running at sprinting speed, prostheses have become so advanced that experts have debated whether they restore [1] or augment [2] performance beyond the biological limb. Counterintuitively, replacing ankle function is more challenging in normal walking than in sprinting. During steady sprint running the biological ankle primarily behaves elastically, whereas in walking at higher speeds the ankle provides net positive work during push-off accounting for about half of the total mechanical work that is performed by the joints of the lower limb in the sagittal plane during a step [3,4]. This aspect of ankle function cannot be entirely replaced by a passive prosthesis.

Although amputation removes muscles that would otherwise consume metabolic energy during walking, people with transtibial amputation typically expend 20 to 30% more metabolic energy than matched able-bodied subjects when using a passive elastic prosthesis [5,6]. This is often attributed to compensations for the lack of net positive ankle push-off work [7]. Higher metabolic rate is typically also accompanied by a lower walking speed and range, which reduces quality of life [8]. Recently, labs and companies have developed powered prostheses that provide net positive work [9-12], which are now becoming commercially available [13].

Robotic prostheses are often designed with the aim of matching the kinematics and kinetics of non-amputee gait [9,14]. Another possible aim would be minimizing metabolic rate for the amputee. The first aim defines an engineering task, in which known biological gait parameters are matched by a robot. The second aim implicitly requires optimization, which could be accomplished through numerical simulation, given a model with predictive validity [15,16], or through systematic exploration of different modes of actuation in experiments [17]. In such a process, it may be advantageous to disregard biological similarity and to explore extreme parameter values, which might even allow amputees to outperform unassisted non-amputees. We recently designed a universal ankle-foot prosthesis emulator [10] that enables this kind of experimental research. The system allows actuation parameters such as ankle push-off work to be varied [18] over a much broader range than with existing commercial prostheses.

Simple dynamic models of walking suggest that the timing of ankle push-off has a substantial effect on metabolic rate. These models contain a step-to-step transition during which the leading leg performs negative ‘collision’ work while the trailing leg performs positive ‘push-off’ work [19-21]. Such models predict that pushing off with the trailing leg starting before leading leg collision reduces energy dissipation, thereby reducing overall mechanical work requirements [19-21]. This prediction is consistent with findings from a recent experiment with a bilateral powered ankle exoskeleton, in which the greatest reduction in metabolic rate was found when exoskeleton push-off started just before leading leg contact [22]. However, in this experiment push-off work was not held constant as timing was varied. We recently found that push-off work affects metabolic rate during walking with prostheses [18] and exoskeletons [23,24]. Another confounding factor was that, because the exoskeleton fits over the biological ankle, subjects were still able to control the timing and magnitude of push-off work of their own biological ankle in parallel with the exoskeleton [25].

**Figure 1:**
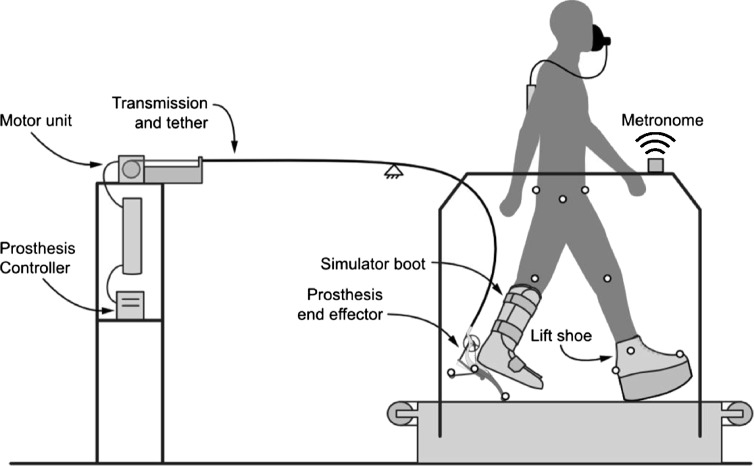
**Experimental setup.** Participants wore a prosthesis attached to a rigid boot and tethered to an off-board motor and control station. To compensate for the leg length difference, subjects wore a lift shoe on their other leg. Step frequency was maintained using a metronome.

The goal of the present study was to measure the isolated effects of timing of total ankle push-off on the energetics and mechanics of walking with a robotic prosthesis. We used a universal ankle-foot prosthesis emulator (Figure 1, Additional file 1: Movie 1) to maintain precise control over total ankle push-off mechanics, and varied the timing of the onset of positive ankle power over a wide range while maintaining constant net prosthesis work. We measured the effects on metabolic rate, center-of-mass mechanics, joint mechanics, and muscle activity. We expected this study to lead to improved understanding of the predictive validity of simple models for walking with a prosthesis, and to inform the design of improved prosthetic limbs.

## Methods

### Participants

We tested 10 able-bodied subjects (60 ± 6 kg, 1.68 ± 0.09 m, 23 ± 2 yr., 6 female and 4 male). The experiment was approved by the Carnegie Mellon University Institutional Review Board, and written informed consent was obtained from all subjects prior to participation.

### Universal ankle-foot prosthesis emulator

Participants walked while wearing an ankle-foot prosthesis emulator [10] on one leg (the 'prosthesis-side leg', Figure 1, Additional file 1: Movie 1). Subjects did not have amputation, and the prosthesis was attached using a rigid boot that immobilized the ankle and weighed 1.9 kg. To compensate for added leg length, subjects wore a lift shoe with a 0.13 m-tall rounded sole weighing 0.9 ± 0.2 kg on their other leg (the “intact leg”). The prosthesis had one degree of freedom in plantarflexion and weighed 1.2 kg. It sensed angle and torque locally, and was tethered to an off-board motor and control station. The emulator was programmed with different behaviors, and had a maximum torque limit of 170 N · m.

### Controller

**Figure 2:**
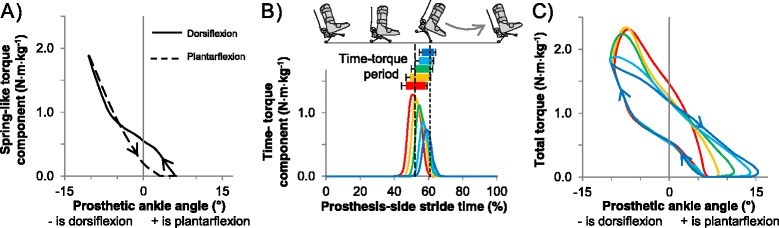
**Prosthesis torque components in timing conditions. (A)** Spring-like torque component, shown in torque-angle space, programmed as a function of prosthesis joint angle (cf. zero-work condition [18]). Solid line is dorsiflexion phase, dashed line is plantarflexion phase. **(B)** Time-torque component, shown in time, programmed as a square wave that started at the desired percent of predicted stride period and lasted 10% of stride period or until toe off. Actual, measured torque increased and decreased gradually. Lines representing earlier torque bursts appear longer than 10% due to averaging of bursts with temporal variation. Bars of later bins are shorter than 10% because the prosthesis leaves the ground. Curve colors correspond to Time-torque onsets. Horizontal bars indicate Time-torque period, and error bars are standard deviation. Vertical dashed lines indicate mean timing of intact-side heel contact and prosthesis toe off. **(C)** Total torque, shown in torque-angle space, was the sum of the Spring-like torque and Time-torque components. Enclosed area is net prosthesis work, which was maintained across timing conditions. Colors are Time-torque bins. All lines and bars represent population means.

In a Spring-like reference condition that mimicked a conventional prosthesis, torque was programmed as a function of prosthesis joint angle (Figure 2A), with stiffness scaled to subject mass. This condition was similar to the zero-work condition in [18].

In push-off timing conditions, the commanded torque was the sum of the Spring-like torque and an additional square wave in time, referred to here as 'Time-torque' (Figure 2). The Time-torque component was programmed to start at a desired percentage of the predicted stride period and to last 10% of the stride period. The controller detected the beginning of each new stride at initial prosthesis forefoot contact. Initial prosthesis heel contact was estimated by subtracting an assumed delay of 10% of the stride between prosthesis heel and forefoot contact (this assumption was not used in post hoc analyses of timing). The stride period of the current stride was predicted using a low-pass filter of previous stride periods:

1 $$ {t}_{sf}(n)=\left(1\hbox{--} \alpha \right)\cdotp {t}_{sf}\left(n\hbox{--} 1\right)+\alpha \cdotp {t}_s(n) $$

where *t*_*sf*_ is filtered stride period, *n* is stride number, *α* is a filter constant (in this case equal to 0.05), and *t*_*s*_ is measured stride period.

The amplitude of the Time-torque square wave was adjusted so as to deliver constant average net positive work per stride per second. The prosthesis work per stride per second was calculated online by multiplying prosthesis torque by prosthesis ankle angular velocity, integrating in time, and dividing by stride period. Filtered work per stride per second was obtained using a similar low-pass filter as with filtered stride time. Time-torque magnitude was adjusted on each stride using an iterative learning approach:

2 $$ {\tau}_t\left(n+1\right)={\tau}_t(n)+{k}_i\cdotp {e}_W(n) $$

where *τ*_*t*_ is the amplitude of the commanded Time-torque square wave, *k*_*i*_ is the iterative learning gain, in this case set to 0.005 N · m · J^−1^ · s, and *e*_*W*_ is the difference between the filtered work per stride per second^a^ and the desired value of 14 J · s^-1^. This value of desired net work strikes a balance between reducing metabolic rate, which decreases with increasing push-off work [18], and increasing the range of viable onset timings, which increases with decreasing push-off work. This value allowed us to explore the timing range where we expected to find an optimum [22].

### Experimental conditions

For each subject we attempted to collect 6 conditions with desired Time-torque onset at 46%, 48%, 50%, 52%, and 54% of the stride period, plus the latest achievable setting between 56% and 60% of the stride period.

Some subjects were unable to adapt to some conditions without the controller reaching torque or velocity limits before achieving 14 J · s^−1^. In such cases, we tried shifting the onset of Time-torque away from the extreme values until we found settings that allowed desired work production. Due to a combination of scheduling constraints and hardware failures, two of the ten subjects walked in only five timing conditions.

### Protocol

Before data collection, subjects completed two adaptation sessions. In the first adaptation session, subjects walked with the prosthesis in three, seven-minute trials. Across trials, subjects were given different timing conditions while treadmill speed was gradually increased to 1.25 m⋅s^−1^, and net push-off work was gradually increased from 0 to 14 J⋅s^−1^.

The second adaptation session started with three minutes of Quiet Standing while resting metabolic rate was measured. In the Normal Walking condition, subjects walked five minutes with normal shoes. Normal Walking was randomly applied either before or after prosthesis conditions. The first prosthesis condition in this adaptation session was the Spring-like condition, during which step frequency was recorded. This frequency was imposed by means of a metronome in all the remaining prosthesis conditions. The Time-torque conditions were presented in random order. The prosthesis conditions lasted seven minutes, and between all conditions subjects were given at least three minutes to rest.

Data were collected in the third session. The protocol of the third session was the same as the second session, except that the order of all prosthesis conditions, including the Spring-like condition, was randomized. All measurements reported in this manuscript are from the third session, except in one subject for whom we report second-day data because the prosthesis malfunctioned at the beginning of the third session. Subjects had at least one full day of rest between sessions.

### Measurements

We measured metabolic rate by means of indirect calorimetry (Oxycon Mobile) during the entirety of each trial. Prosthesis sensor data were recorded for the last 2.5 minutes at a rate of 500 Hz using prosthesis control software. All other measurements were taken over 30-second periods at the end of each trial. Ground reaction forces were measured at a rate of 2000 Hz by force plates built into a split-belt treadmill (Bertec). Muscle activity was measured for the primary, accessible leg flexors and extensors (*rectus femoris, vastus medialis,* and *biceps femoris* in both legs; and *gastrocnemius medialis* and *lateralis, soleus,* and *tibialis anterior* in the intact-side leg) at a rate of 2000 Hz with wireless surface electromyography sensors (Delsys). Joint kinematics were measured at a rate of 100 Hz by a camera-based motion capture system (Vicon) tracking a set of 17 reflective markers on the prosthesis, lower limbs, and pelvis, and recorded in motion capture software along with ground reaction forces and electromyography data. At the end of each Time-torque condition, subjects scored their perception of the condition compared to the Spring-like condition on a scale of −10 to +10 where −10 was ‘cannot walk’ and +10 was ‘walking is effortless’.

### Data processing

Metabolic rate was calculated from mean $\dot{\mathrm{V}}$ O_2_ and $\dot{\mathrm{V}}$ CO_2_ values [26] of the last three minutes of each walking condition, and the last two minutes of the resting condition.

Prosthesis mechanics were calculated from onboard sensor data. Signals were filtered with a second-order Butterworth low-pass filter with a cutoff frequency of 100 Hz. Prosthesis power was calculated for the 2.5 minute recording period. Net work per unit time was calculated by integrating prosthesis power in time over the whole stance phase and dividing by stride period.

Motion capture data were first gap-filled in Vicon Nexus and then remaining gaps were reconstructed in Visual3D based on other markers on the same segment. For joint kinetic and kinematic analyses, marker and ground reaction force signals were filtered with a second-order Butterworth low-pass filter with a 15 Hz cutoff frequency. Three-dimensional inverse dynamics analysis was performed and sagittal plane joint angles, torques and powers were calculated using a custom script in Matlab.

In a separate analysis, we used ground reaction forces to calculate center-of-mass work. Ground reaction force signals were low-pass filtered at 30 Hz with a second-order Butterworth filter. Center-of-mass velocity was calculated by dividing total ground reaction force by body mass and integrating in time. Center-of-mass power for each leg was calculated according to the individual limbs method [27]. We calculated center-of-mass push-off work (positive prosthesis-side work between approximately 45% and 65% of prosthesis stride), collision work (negative intact-side work between about 45% and 65% of prosthesis stride) and rebound work (positive intact-side work between about 65% and 80% of prosthesis stride). We also analyzed the maximum vertical ground reaction force during the first half of stance phase as an indication of the potential effect on the risk for overuse injuries in the intact limb [28-30].

Electromyography signals were filtered with a 50–400 Hz band-pass filter, rectified, and filtered with a root mean square filter with a 0.1 s moving window. Based on visual inspection, 69 signals out of a total of 624 trial-muscle combinations were identified as containing inaccurate electromyography data and were removed from analysis. Erroneous signals were characterized by uncorrelated, saturating spikes, which were likely the result of electrodes becoming dislodged. We calculated maximum values during periods of interest, such as prosthesis-side push-off.

Although the online prosthesis timing control was based on prosthesis forefoot contact, during post hoc analysis time series data were stride-normalized using prosthesis heel contact detected from unfiltered ground reaction forces. Step length was calculated by dividing step time by the treadmill speed. Step width was calculated by means of the lateral distance between the positions of foot markers during subsequent stance phases. In timing conditions, the actual onset of Time-torque was determined by detecting the maximum, unfiltered second derivative of the residual of Total-torque minus Spring-like torque. The ending of push-off was detected as the instant the measured prosthesis torque became zero. All metrics were calculated on each stride before averaging, except for joint kinetics which were calculated based on an average stride.

### Data organization

**Figure 3:**
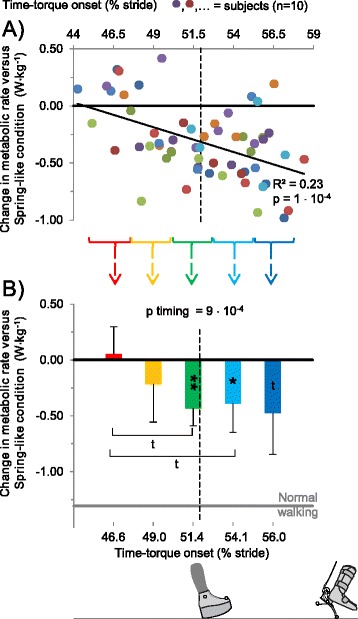
**Change in metabolic rate versus Time-torque onset. (A)** Change in metabolic rate with respect to the Spring-like condition versus Time-torque onset. Colors are different subjects. Thin solid black line shows linear regression. Curly brackets indicate borders of Time-torque bins. **(B)** Change in metabolic rate for each Time-torque bin. Bar colors correspond to Time-torque onsets. Horizontal black line is mean for Spring-like condition. Gray line is mean for Normal Walking. Vertical dashed lines represent mean timing of intact-side heel contact. Error bars are inter-subject standard deviations. P-values are from repeated measures ANOVA on timing bins. Symbols inside bars indicate significant differences versus Zero-work estimate. Brackets with symbols represent pairwise differences between timing conditions. ** = p ≤ 0.01, * = p ≤ 0.05, t = p ≤ 0.1.

In order to allow visualization of population averages and statistical comparisons despite inter-subject differences in actual Time-torque onset, timing trials were grouped into five Time-torque onset bins with borders at 45.1%, 47.6%, 50.1%, 52.6%, 55.2% and 57.7% of the stride cycle, referred to as Earliest, Early, Middle, Late, and Latest respectively (Figure 3). Despite careful choice of these bins, there were four trials that fell outside the included ranges. These outliers occurred due to differences between online and post hoc timing, and due to the limited capacity of the prosthesis to apply push-off very early or very late in stance while maintaining desired work.

### Statistics

We tested the effect of push-off timing by means of a repeated-measures analysis of variance including only the timing conditions. If the p-value was below 0.1, pairwise comparisons were made by means of paired t-tests.

To compare timing conditions to the Spring-like condition, we first checked for an overall effect by means of repeated-measures analysis of variance, this time including the Spring-like condition. If the p-value was below 0.1, we checked for differences between timing conditions and the Spring-like condition using paired t-tests. In pairwise tests we corrected for multiple comparisons with a Šídák-Holm correction [31].

Perception scores in the Time-torque conditions and net prosthesis work in the Spring-like condition were compared relative to zero with unpaired t-tests. We calculated Pearson’s correlation between metabolic rate and Time-torque onset. In all analyses we used a significance level of α = 0.05.

## Results

### Prosthesis mechanics

**Figure 4:**
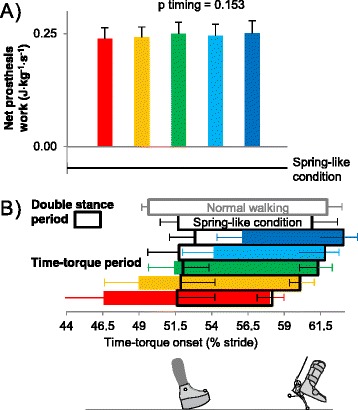
**Prosthesis push-off work and timing. (A)** Net prosthesis work did not vary across timing conditions. Horizontal black line is Spring-like condition. P-value is from repeated measures ANOVA on timing bins. **(B)** Double support and Time-torque periods. Bar colors correspond to Time-torque onsets. Error bars are standard deviations.

Time-torque onsets ranged from 44.3 to 58.4% of stride period (Figure 3A). The average onset timings of the five Time-torque bins were 46.6 ± 0.7 (Earliest bin), 49.0 ± 0.7 (Early), 51.4 ± 0.7 (Middle), 54.1 ± 0.7 (Late) and 56.0 ± 0.7% (Latest) of stride (mean ± standard deviation; Figure 4B).

**Figure 5:**
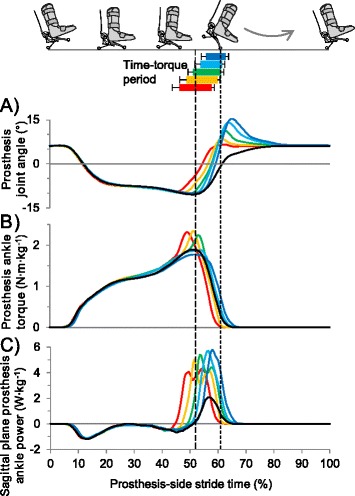
**Prosthesis sensor data versus stride time. (A)** Prosthetic joint angle in time (normalized to stride period). Stride period in this and other time-series charts begins and ends at prosthesis heel contact. **(B)** Total prosthesis torque (i.e. Spring-torque + Time-torque). **(C)** Prosthetic ankle power. Curve colors correspond to Time-torque onsets. Black curve is Spring-like condition. Horizontal bars indicate Time-torque period. Vertical dashed lines indicate mean timing of intact-side heel contact and prosthesis toe off.

Although Time-torque was commanded as a square wave, the resulting mean trajectory increased and decreased smoothly (Figure 2B). Recursive adjustment of torque to maintain constant work led to progressively lower peak Time-torque values with later Time-torque onsets. Total prosthesis peak torque was lower with later push-off timing (p = 1 · 10^−5^, ANOVA; Figure 5B). Later Time-torque onset led to later prosthesis positive power onset, as expected (p = 2 · 10^−11^, ANOVA; Figure 5). Mean prosthesis power had two peaks in the Earliest to Middle Time-torque bins, whereas there was only one peak in the Late and Latest bin. Negative prosthesis ankle work did not change across timing conditions (p = 0.6, ANOVA).

Mean net prosthesis work in Time-torque conditions was 0.25 ± 0.03 J · kg^−1^ · s^−1^ (Figure 4A). Net prosthesis work did not change significantly with timing (p = 0.2, ANOVA). In the Spring-like condition, net push-off work was lower than zero (−0.05 ± 0.02 J · kg^−1^ · s^−1^, p = 4 · 10^−21^, unpaired t-test) characterized by hysteresis of 23 ± 5%.

### Metabolics

Metabolic rate was inversely correlated with Time-torque onset (R^2^ = 0.23, p = 1 · 10^−4^, Pearson’s correlation; Figure 3A). Net metabolic rate in the Earliest through Latest Time-torque bins was 4.60 ± 0.87, 4.32 ± 0.98, 4.04 ± 0.81, 4.11 ± 0.91 and 4.02 ± 1.04 W · kg^−1^, respectively (Figure 3B). Net metabolic rate in the Spring-like condition was 4.47 ± 0.73 W · kg^−1^. In the Earliest Time-torque bin, metabolic rate appeared to be about 0.06 ± 0.24 W · kg^−1^ (1 ± 6%) higher than in the Spring-like condition whereas in the Middle and Late Time-torque bins metabolic rate was on average 0.41 ± 0.20 W · kg^−1^ (10 ± 7%) lower^b^ than the Spring-like condition (p = 1 · 10^−4^, 0.025, respectively, paired t-tests; Figure 3B). Metabolic rate in the Latest Time-torque bin showed a trend towards being lower than the Spring-like condition (p = 0.06, paired t-test).

Net metabolic rate during Normal Walking was 3.17 ± 0.50 W · kg^−1^. This was 0.87 ± 0.54 W · kg^−1^ (20 ± 10%) lower than in the Middle Time-torque bin (p = 0.01, paired t-test).

### Center-of-mass mechanics

**Figure 6:**
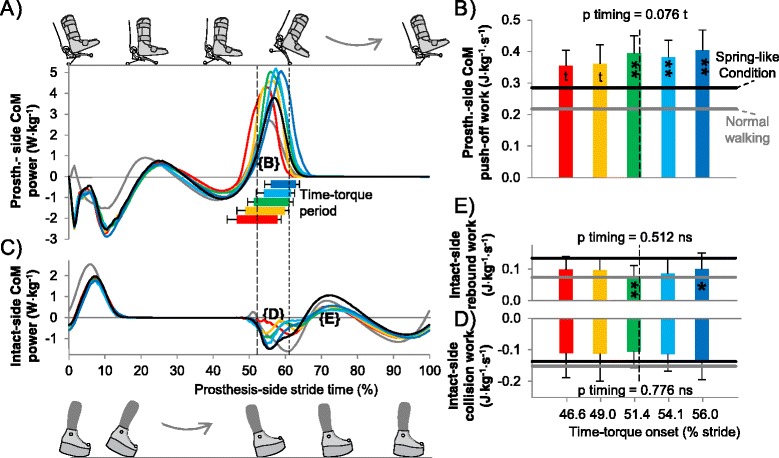
**Center-of-mass power and work. (A)** Prosthesis-side center-of-mass power. **(B)** Prosthesis-side push-off work. **(C)** Intact-side center-of-mass push-off power. **(D)** Intact-side collision work. **(E)** Intact-side rebound work. Bar and curve colors correspond to Time-torque onsets. Black line is Spring-like condition. Gray line is Normal Walking. Horizontal bars indicate Time-torque periods. Vertical dashed lines indicate mean timing of intact-side heel contact and prosthesis toe-off. Error bars are inter-subject standard deviation. P-values are from repeated measures ANOVA on timing bins. Symbols inside bars indicate significant differences from Spring-like condition. ** = p ≤ 0.01, * = p ≤ 0.05, t = p ≤ 0.1.

The onset of prosthesis-side positive center-of-mass push-off work occurred later in the stride period with later onset of Time-torque (p = 0.005, ANOVA; Figure 6A). There was also a trend towards higher prosthesis-side center-of-mass push-off work with later Time-torque onset (p = 0.08, ANOVA; Figure 6B). Prosthesis-side center-of-mass push-off work in the Middle to Latest Condition was higher than the Spring-like condition (p < 5 · 10^−4^, paired t-tests) which was in turn higher than in Normal Walking (p = 2 · 10^−5^, paired t-test).

Time-torque onset appeared to influence the time course of intact-side collision power (Figure 6C) but did not have a significant effect on collision work (p = 0.8, ANOVA; Figure 6D). The intact-side maximum ground reaction force during the first half of stance decreased with later push-off timing (p = 0.017, ANOVA), from 123 ± 13% of body weight in the Earliest Time-torque bin to 111 ± 6% of body weight in the Latest Time-torque bin (Additional file 2: Figure S1C). However, center-of-mass velocity appeared to increase with later Time-torque onsets (Additional file 2: Figure S1E). There were no significant differences in intact-side collision work between the Time-torque bins and the Spring-like condition (p > 0.45, paired t-tests) and there was no significant difference in intact-side collision work between the Spring-like condition and Normal Walking (p = 0.41, paired t-test).

Time-torque onset did not affect intact-side rebound work (p = 0.5, ANOVA), but in the Middle and Latest Time-torque bins, rebound work was smaller than in the Spring-like condition (p = 0.006 and p = 0.041, respectively, paired t-tests, Figure 6E). Rebound-work in the Spring-like condition was higher than in Normal Walking (p = 2 · 10^−4^, paired t-test).

### Electromyography

**Figure 7:**
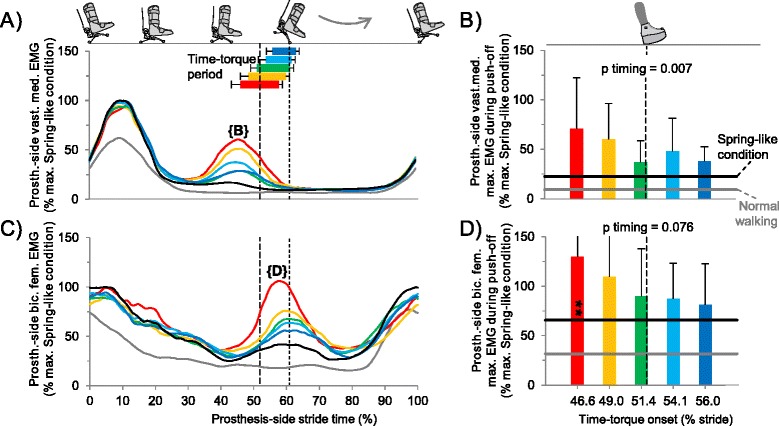
**Electromyography. (A)** Prosthesis-side average *vastus medialis* electromyograms (EMG). **(B)** Peak *vastus medialis* EMG during late stance (30-60% stride). **(C)** Prosthesis-side average *biceps femoris* EMG. **(D)** Peak *biceps femoris* EMG during late stance. Bar and curve colors correspond to Time-torque onsets. Black line is Spring-like condition. Gray line is Normal Walking. Horizontal bars indicate Time-torque period. Vertical dashed lines represent mean timing of intact-side heel contact and prosthesis toe off. Error bars are inter-subject standard deviations. P-values are from repeated measures ANOVA on timing bins. Symbols inside bars indicate significant differences versus Spring-like condition. ** = p ≤ 0.01, * = p ≤ 0.05, t = p ≤ 0.1.

We observed activation bursts at around 45% of the stride cycle in the *vastus medialis* (Figure 7A) and at around 60% of the stride in the *biceps femoris* (Figure 7C) in the prosthesis-side leg during Time-torque conditions, but not during Normal Walking. Earlier Time-torque onset led to higher peak values in this burst in *vastus medials* activity (p = 0.007, ANOVA; Figure 7B) and led to a trend towards higher peak values in the burst in *biceps femoris* activity (p = 0.08 ANOVA; Figure 7D). The *biceps femoris* peak activation in the Earliest Time-torque bin was higher than in the Spring-like condition (p = 0.008, paired t-test). Peak activation in other muscles around the time of prosthesis push-off did not show a clear relationship with Time-torque onset (p > 0.106, ANOVA, Additional file 3: Figure S2).

### Joint kinetics

**Figure 8:**
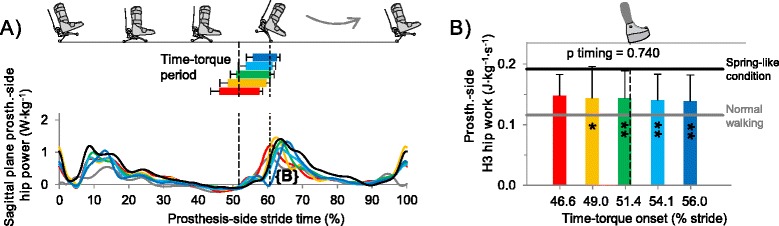
**Hip joint power and work. (A)** Prosthesis-side hip power. **(B)** Prosthesis-side hip positive work during swing initiation (H3, 50-90% stride). Bar and curve colors correspond to Time-torque onsets. Black line is Spring-like condition. Gray line is Normal Walking. Horizontal bars indicate Time-torque period. Vertical dashed lines represent mean timing of intact-side heel contact and prosthesis toe off. Error bars are inter-subject standard deviations. P-values are from repeated measures ANOVA on timing bins. Symbols inside bars represent significant differences versus Spring-like condition. ** = p ≤ 0.01, * = p ≤ 0.05, t = p ≤ 0.1.

Prosthesis-side ankle push-off work, calculated from inverse dynamics, was on average 1.7 times higher in the Time-torque conditions than during Normal Walking (p < 3 · 10^−3^, paired t-tests, Additional file 4: Figure S3). Hip joint power in the prosthesis-side leg associated with swing initiation [3] appeared to shift later with later Time-torque onset (Figure 8A). Hip work during this period did not differ across Time-torque bins (p = 0.7, ANOVA), but work was lower than in the Spring-like condition for all Time-torque onsets other than in the Earliest bin (p < 0.047, paired t-tests; Figure 8B). There were no clear effects of push-off timing on any other outcomes in joint work around the time of prosthesis push-off (Additional file 4: Figure S3).

### Spatiotemporal and variability measures

During prosthesis conditions, intact-side heel contact occurred at 52.0 ± 1.9% of stride, whereas in Normal Walking intact-side leg heel contact occurred at 49.6 ± 0.5% of stride (Figure 8A). There was no effect of Time-torque onset on the timing of intact-leg contact (p = 0.3, ANOVA), but later Time-torque onset led to longer prosthesis-side stance duration (p = 1 · 10^−9^, ANOVA).

**Figure 9:**
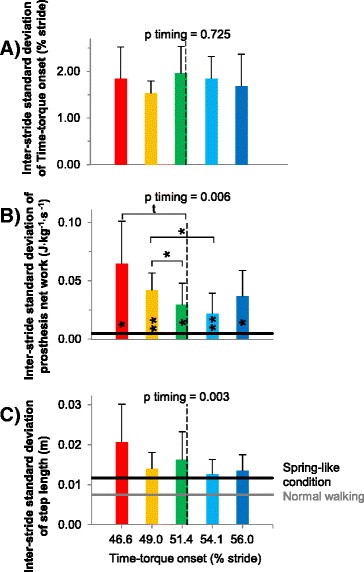
**Variability of prosthesis timing, prosthesis work and step length. (A)** Inter-stride standard deviation in Time-torque onset time. **(B)** Inter-stride standard deviation in prosthesis net work. **(C)** Inter-stride standard deviation of step length. Colors correspond to Time-torque bins. Horizontal black line is Spring-like condition. Gray line is Normal Walking. Vertical dashed line indicates mean timing of intact-side heel contact. Error bars are standard deviations of inter-stride standard deviations. P-values are from repeated measures ANOVA on timing bins. Symbols inside bars are significant differences versus Spring-like condition. Brackets with symbols indicate pairwise differences between timing conditions. ** = p ≤ 0.01, * = p ≤ 0.05, t = p ≤ 0.1.

There were no differences in inter-stride variability of Time-torque onset between the Time-torque bins (p = 0.7, ANOVA; Figure 9A). However, earlier Time-torque onset led to increased inter-stride variability in net prosthesis work (p = 0.006, ANOVA; Figure 9B). For example, prosthesis work variability was about twice as high in the Early onset bin compared to the Late onset bin (p = 0.04, paired t-test). In all Time-torque bins except the Late bin, push-off work variability was higher than in the Spring-like condition (p < 0.031, paired t-tests). Earlier Time-torque onset also led to increased inter-stride variability in step length (p = 0.003, ANOVA; Figure 9C).

### Perception

Time-torque onset had a significant effect on perception score (p = 3 · 10^−4^, ANOVA; Additional file 5: Figure S4). The Medium to Latest timing bins were perceived to be more assistive than the Spring-like condition (p < 0.02, unpaired t-tests) and the perception score in the Latest Time-torque bin was better than in the Earliest Time-torque bin (p = 2 · 10^−4^, paired-t test).

## Discussion

The Timing of the onset of prosthetic ankle push-off had a substantial effect on metabolic rate. In the Latest push-off timing condition, metabolic rate was about 10% lower than in the Earliest condition, a difference that is equivalent to reductions that have been found when using autonomous robotic prostheses compared to passive ones [11]. The Spring-like condition approximated the stiffness and hysteresis of typical passive prostheses [32]. When net push-off work equivalent to almost twice the value in Normal Walking was provided with the Earliest timing, it did not reduce metabolic rate. Adding motors and batteries to a passive prosthesis therefore might not benefit the user if the timing of motor actuation is not appropriate. It has been posited that reductions in metabolic rate with robotic devices are proportional to positive device work and peripheral mass [33], but other factors, such as timing, seem to play an important role as well.

During Normal Walking, the onset of positive biological ankle joint work occurred at 49.6% of the stride cycle (Additional file 4: Figure S3 G,P). It is known that during Normal Walking the onset of positive ankle joint work depends on walking speed [14]. In this study, participants’ legs were effectively lengthened from 0.92 m to 1.05 m by the lift shoe and prosthesis, lowering their non-dimensional speed. In Normal Walking, the effect of an equivalent decrease in non-dimensional walking speed could explain about 1% later push-off timing [14]. During prosthesis walking, the intact-side heel strike occurred at about 52% stride rather than 50% (an asymmetry that could have been even more pronounced if the subjects were not instructed to walk to the beat of a metronome). The Middle timing condition, centered on 51.4% stride, is therefore consistent with push-off timing in Normal Walking. In this sense, push-off that began at or after the normal time led to reduced energy cost.

The simplest walking model predicts that impulsive push-off just before leading leg contact minimizes mechanical work requirements [19]. However, in the simplest model collision is an instantaneous event as opposed to actual walking where collision is a phase with negative center-of-mass work that is spread out in time. More recent models with a double stance phase show that push-off throughout double stance reduces collision losses [20,21]. Still, these models predict that optimal push-off should start before intact-side heel strike, and that as push-off occurs earlier, center-of-mass collision work should be reduced. These models sometimes do not include certain features of humans [34], such as multi-segmented legs and biological muscle properties, which could explain the differences between their predictions and the present findings.

A recent study with ankle exoskeletons reported optimal push-off onsets at around 45% of stride [22], and another case study reported optimal onset at 30% of stride [35]. However, exoskeletons operate in parallel with the limb, rather than in series, and therefore do not constrain total-joint push-off onset. Prior experiments on exoskeleton push-off timing have also not maintained constant work across conditions, and earlier actuator onset may have resulted in more net work by the device. Exoskeletons have typically also been applied bilaterally, whereas the unilateral prosthesis used here resulted in some asymmetry. It is therefore difficult to compare the results of these studies.

Our finding is consistent, on the other hand, with an anecdotal report by one subject with amputation in a different study that stated that ‘the best timing for adding power was when the heel of the adjacent foot had initial contact’ [12]. Amputation may lead to changes in morphology that interact with push-off timing, leading to later optima.

The absence of a reduction in metabolic rate when push-off work was provided early in stance may be explained by increased thigh muscle activity prior to and during push-off. Increased prosthesis-side *vastus* EMG at about 45% stride in conditions with early push-off (Figure 7A,B) might have prevented undesirable knee flexion during the ensuing push-off, resisting high limb loads generated by the prosthesis to maintain desired work (Figure 5B). A similar mechanism was suspected for amputees using an energy recycling prosthesis [36]. Increased prosthesis-side *biceps femoris* EMG at about 60% stride in conditions with early push-off (Figure 7C,D) might have acted to flex the knee during the ensuing swing phase, providing an effect similar to that attributed to the biarticular *gastrocnemius* during normal gait. This increase might also have led to stabilizing co-activation at the knee during the period of stance in which both *biceps femoris* and *vastus* activity was increased. Thigh muscles might also have acted to dissipate some of the work performed by the prosthesis in early push-off conditions, although measured absorption at the knee joint during this period did not seem to change with timing. Taking in consideration the high muscle volume of the thigh muscles it is possible that increased prosthesis-side thigh muscle activity, perhaps in combination with apparent trends in other muscle activity, may explain the differences in metabolic rate between the Earliest and Latest push-off onset conditions.

The effects of prosthesis behavior on gait stability may also help explain the lack of a benefit with preemptive push-off. With early push-off timing, we found increases in inter-stride variability in both push-off work and step length (Figure 9B,C). Since the prosthesis control algorithm remained the same, the observed increases in variability must have come from changes in the human walking with it. Some amount of variability is natural during human gait [37,38], but these increases, without any changes to subject physiology or environmental conditions, likely indicate increases in step-by-step control actions related to balance, which can be associated with higher metabolic rate [38,39]. Increased variability might stem from difficulty in controlling the effects of prosthesis forces during early push-off conditions, consistent with the observed increased activation of knee musculature. It may have been easier to control the flow of work from the trailing leg once the leading leg was placed on the ground, affording, e.g., improved means for regulating net impulse on the center of mass. This echoes anecdotes from two simple walking robots [40,41], for which preemptive push-off seemed to result in poorer stability. Longer double-support periods, as were found with later push-off timing (Figure 4B), would similarly increase the time during which both limbs could be used to stabilize body motions.

Energy use associated with leg swing initiation did not seem to explain the increased benefits of push-off work with later timing. Some portion of late push-off work may go towards acceleration of the swing leg [42], and this requirement is likely greater with the added mass of the simulator boot and prosthesis (2.9 kg). However, although we observed later onset of H3 hip power with later push-off, we did not observe a reduction in total hip work (Figure 8B).

The observed relationships between push-off timing, mechanical work and metabolic rate were not consistent with predictions from simple dynamic models of walking. Such models predict that preemptive trailing leg push-off will reduce collision dissipation in the leading leg, which did not occur here (Figure 6D). With early push-off work we also did not find reductions in the intact-side maximum vertical ground reaction force during the first half of stance. This could be due to a similar mechanism as in another prosthesis study where increased intact-side vertical ground reactions were found with prostheses with shorter forefoot rockers [28]. Active prosthesis push-off has previously been observed to decrease the intact-side maximum vertical ground reaction force during the first half of stance [18,29,30]. Intact-side maximum vertical ground reaction force during the first halve of stance decreased with later onset of push-off, the opposite of what one might expect from simple models, but this change was offset by increases in magnitude of the vertical component of center-of-mass velocity, consistent with [18]. Joint work in the intact-side leg during collision also did not appear to be affected by push-off timing. Simple dynamic models of walking also make predictions for energy use based on the assumption that changes in metabolic rate will be correlated to changes in center-of-mass work, which was not the case here (Figure 3B vs. Additional file 6: Figure S5B). Another study with a robotic prosthesis did find a correlation between these outcomes [11], but made comparisons across two prostheses with other mechanical differences that could have affected center-of-mass work.

Our findings are limited by the way in which we enforced push-off timing and the measurements we made of human response. The higher-than-normal prosthesis work we imposed and the use of the simulator boot prevent us from making strong claims about optimal timing in Normal Walking. However, we do believe that the experiment was appropriate as a test of simple model predictions, since the normal phases in center-off-mass power were all present including a phase with net negative center-of-mass collision work in the intact-side leg. Prosthesis parameters that were not held constant across timing conditions might have had confounding effects, although we could not identify them. For example, we observed higher peak torque and a longer (less impulsive) double-peaked power in earlier push-off conditions. Higher peak torque and double-peaked power do not explain higher metabolic rate in Earliest and Early timing conditions, however, since the Middle timing condition also contained these features (Figure 5) but substantially reduced metabolic rate (Figure 3B). It also does not seem that prosthesis push-off impulsiveness in the earlier Time-torque bin explains the lack of reduction in metabolic rate, since the vertical component of center-of-mass velocity was higher (more re-directed) at the onset of leading leg contact with earlier Time-torque onset (Additional file 2: Figure S1E). Other aspects of the intervention might have affected our results, of course. A more gradual Time-torque profile, or other control technique, could have generated smoother total prosthesis torque and power trajectories. Perhaps this would have been easier for the user to control, leading to less co-activation and less effort in conditions with early push-off timing.

In statistical analyses of candidate metabolic cost correlates we only looked at center-of-mass energetics, work bursts of sagittal-plane joint kinetics and peak activations of the main flexor and extensor muscles around the prosthesis push-off phase. It could be that differences in metabolic rate could be better explained with frontal-plane kinetics, translational work in the hip joint [43] or muscle activations of mono-articular hip muscles. Measurements were also not taken inside the prosthesis boot, and it is possible that participants compensated for device behavior by, e.g., using their ankle to damp out some work during early-onset push-off conditions. We also did not measure muscle fascicle kinematics, which can explain changes in metabolic rate that are not visible at the joint level, including changes caused by external devices [44].

The timing of push-off can be defined with respect to several events in the gait cycle. We chose to present our results versus prosthetic heel contact because they were more evenly distributed this way, likely because the online controller timed push-off based on prosthesis forefoot contact. We also prefer this reference point because it is more relevant to prosthesis design, since prostheses are typically controlled based on internal sensors. Simple model predictions of the effects of push-off timing are usually made with respect to the instant of collision of the leading leg, which is modeled to occur instantaneously at leading leg heel strike in such models. We also analyzed metabolic data using timing with respect to heel strike (and several other events) and found nearly identical mean results.

These results might not translate to individuals with amputation. Amputees have more experience walking with (particular types of) prostheses than our subjects and may have more compliance at the socket interface [36]. The effects of push-off timing might be influenced by the added mass and leg length of the simulator boot, which would not be present for amputees. We have previously observed opposite effects of intervention between these populations [36], and so tests among participants with amputation must be performed before implications for device design can be addressed directly.

## Conclusions

Prosthesis push-off timing, isolated from push-off work, had a strong effect on metabolic rate, with optimal push-off occurring at or after opposite-leg heel contact. Neither the optimal timing nor the effects on collision work were consistent with predictions from simple dynamic models of walking. Optimal push-off timing in this unilateral prosthesis was also later than observed for bilateral exoskeletons, which may be related to asymmetry or differences in regulation of net joint work. With early push-off, potential benefits of net prosthesis work input seem to have been offset by a combination of increased balance-related effort and increased muscle activity in the prosthesis-side knee flexors and extensors during push-off. With later push-off, benefits compared to the Spring-like condition seem to have been partially derived from reduced muscle activity at the hip during swing initiation. It therefore appears that push-off before intact-side heel strike may not be advantageous in ankle-foot prostheses, although studies among individuals with amputation are needed to verify this idea.

### Endnotes

^a^Work measurements are normalized versus stride period in order to facilitate comparison with metabolic rate measurements. While the J · s^−1^ could be converted into W, we chose to present work measurements in J · s^−1^ in order to keep a clear distinction from measurements of instantaneous power.

^b^Small discrepancies between reported mean values of Time-torque bins and mean differences between Time-torque bins are due to missing trials due to unanticipated differences in the controlled timing and actual timing, hardware limitations in the extreme timing conditions and scheduling constraints.

## Additional files

Additional file 1: Movie 1. Walking with the robotic prosthesis. Participant walking on a treadmill with the prosthesis in different timing conditions. Prosthesis worn on the right leg and the lift shoe worn on the left leg. Additional file 2: Figure S1. Ground reaction forces and center-of-mass-velocity. (A) Prosthesis-side vertical ground reaction force. (B) Prosthesis-side anterior-posterior ground reaction force. (C) Intact-side vertical ground reaction force. (D) Intact-side anterior-posterior ground reaction force. (E) Vertical component of center-of-mass velocity. (F) Anterior-posterior component of center-of-mass velocity. Bar and curve colors correspond to Time-torque onsets. Black line is Spring-like condition. Gray line is Normal Walking. Horizontal bars indicate Time-torque period. Vertical dashed lines represent mean timing of intact-side heel contact and prosthesis toe off. Additional file 3: Figure S2. Electromyography. (A-C) Prosthesis-side *rectus femoris*, *vastus medialis* and *biceps femoris* electromyograms. (D-K) Intact-side *rectus femoris*, *vastus medialis*, *biceps femoris*, *tibialis anterior*, *gastrocnemius lateralis*, *gastrocnemius medialis*, *soleus lateralis*, and *soleus medialis* electromyograms. Bar and curve colors correspond to Time-torque onsets. Black line is Spring-like condition. Gray line is Normal Walking. Horizontal bars indicate Time-torque period. Vertical dashed lines indicate mean timing of intact-side heel contact and prosthesis toe off. Additional file 4: Figure S3. Sagittal-plane joint kinematics and kinetics. (A to C) Prosthesis-side joint angles. (D to F) Prosthesis-side joint moments. (G to I) Prosthesis-side joint powers. (J to L) Intact-side joint angles. (M to O) Intact-side moments. (P to R) Intact-side joint powers. Bar and curve colors correspond to Time-torque onsets. Black line is Spring-like condition. Gray line is Normal Walking. Horizontal bars indicate Time-torque period. Vertical dashed lines indicate mean timing of intact-side heel contact and prosthesis toe off. Additional file 5: Figure S4. Perception scores. Mean perception scores for each Time-torque bin compared to the Spring-like condition. Preference was reported on a scale from −10 to +10, where −10 was ‘cannot walk’ and +10 was ‘walking is effortless’. Error bars are inter-subject standard deviations. P-value is from a repeated measures ANOVA on timing bins. Symbols inside bars represent significant differences versus Spring-like condition. Brackets represent pair-wise differences between conditions. ** = p ≤ 0.01, * = p ≤ 0.05, t = p ≤ 0.1. Additional file 6: Figure S5. Metabolic rate estimates. (A) Estimated metabolic rate based on center-of-mass power, which implicitly assumes all work is performed by muscle fascicles. The biological component of prosthesis-side center-of-mass power was obtained by subtracting prosthesis power from prosthesis-side center-of-mass power. Positive and negative power phases were then divided by their theoretical muscle efficiencies (25% and −120%, respectively). The same process was applied to intact-side center-of-mass power, and the two sides were then added together. (B) Estimated average metabolic rate based on center-of-mass power, assuming that all work is performed by muscle fascicles. (C) Estimated metabolic rate based on total joint power, obtained by dividing positive and negative joint power regions by their respective theoretical muscle efficiencies (25% and −120%, respectively) and adding the resulting trajectories for all human joints. (D) Average estimated metabolic rate based on total joint power. Bar and curve colors correspond to Time-torque onsets. Black line is Spring-like condition. Gray line is Normal Walking. Horizontal bars indicate Time-torque periods. Vertical dashed lines indicate mean timing of intact-side heel contact and prosthesis toe-off. Error bars are inter-subject standard deviation. P-values are from repeated measures ANOVA on timing bins. Symbols inside bars indicate significant differences from Spring-like condition. ** = p ≤ 0.01, * = p ≤ 0.05, t = p ≤ 0.1.
