# Supplementary figures and images for: The influence of push-off timing in a robotic ankle-foot prosthesis on the energetics and mechanics of walking
Source: J Neuroeng Rehabil. 2015 Feb 22;12:21. doi: 10.1186/s12984-015-0014-8 (PMC4404655; doi:10.1186/s12984-015-0014-8)

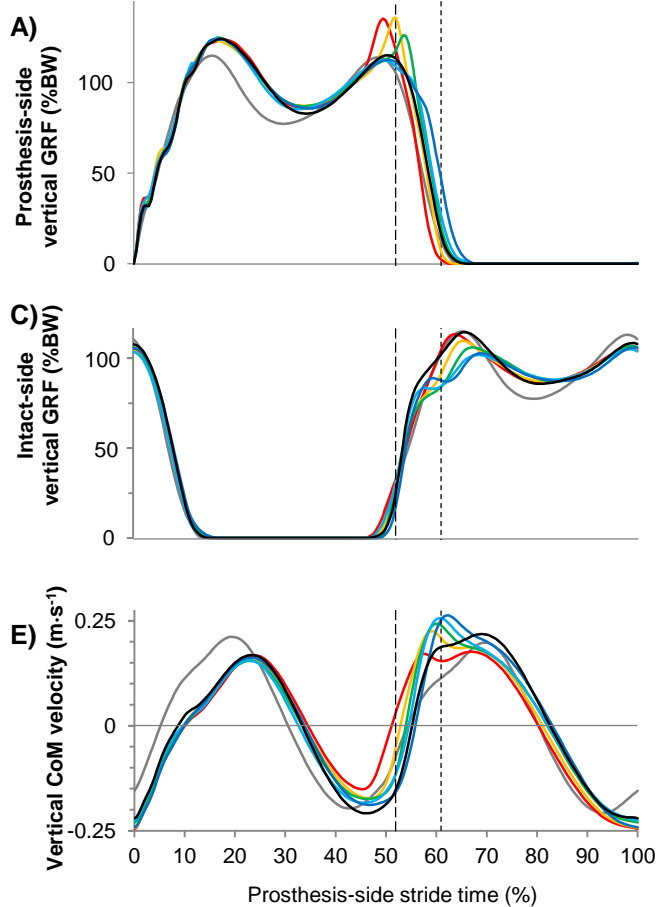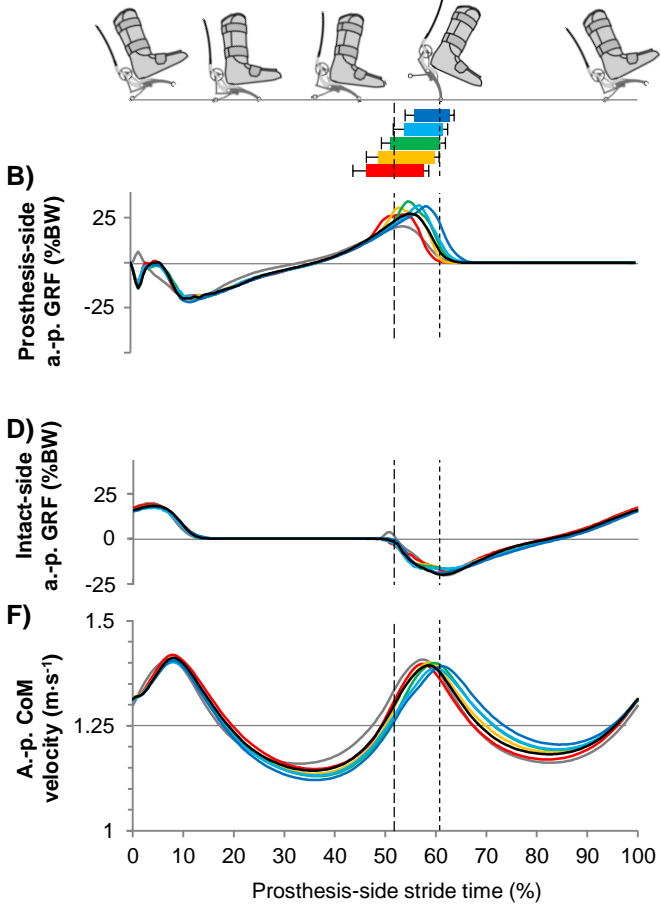

Supplement: Additional file 2: Figure S1. — Ground reaction forces and center-of-mass-velocity. (A) Prosthesis-side vertical ground reaction force. (B) Prosthesis-side anterior-posterior ground reaction force. (C) Intact-side vertical ground reaction force. (D) Intact-side anterior-posterior ground reaction force. (E) Vertical component of center-of-mass velocity. (F) Anterior-posterior component of center-of-mass velocity. Bar and curve colors correspond to Time-torque onsets. Black line is Spring-like condition. Gray line is Normal Walking. Horizontal bars indicate Time-torque period. Vertical dashed lines represent mean timing of intact-side heel contact and prosthesis toe off. [file 12984_2015_14_MOESM2_ESM.pdf]

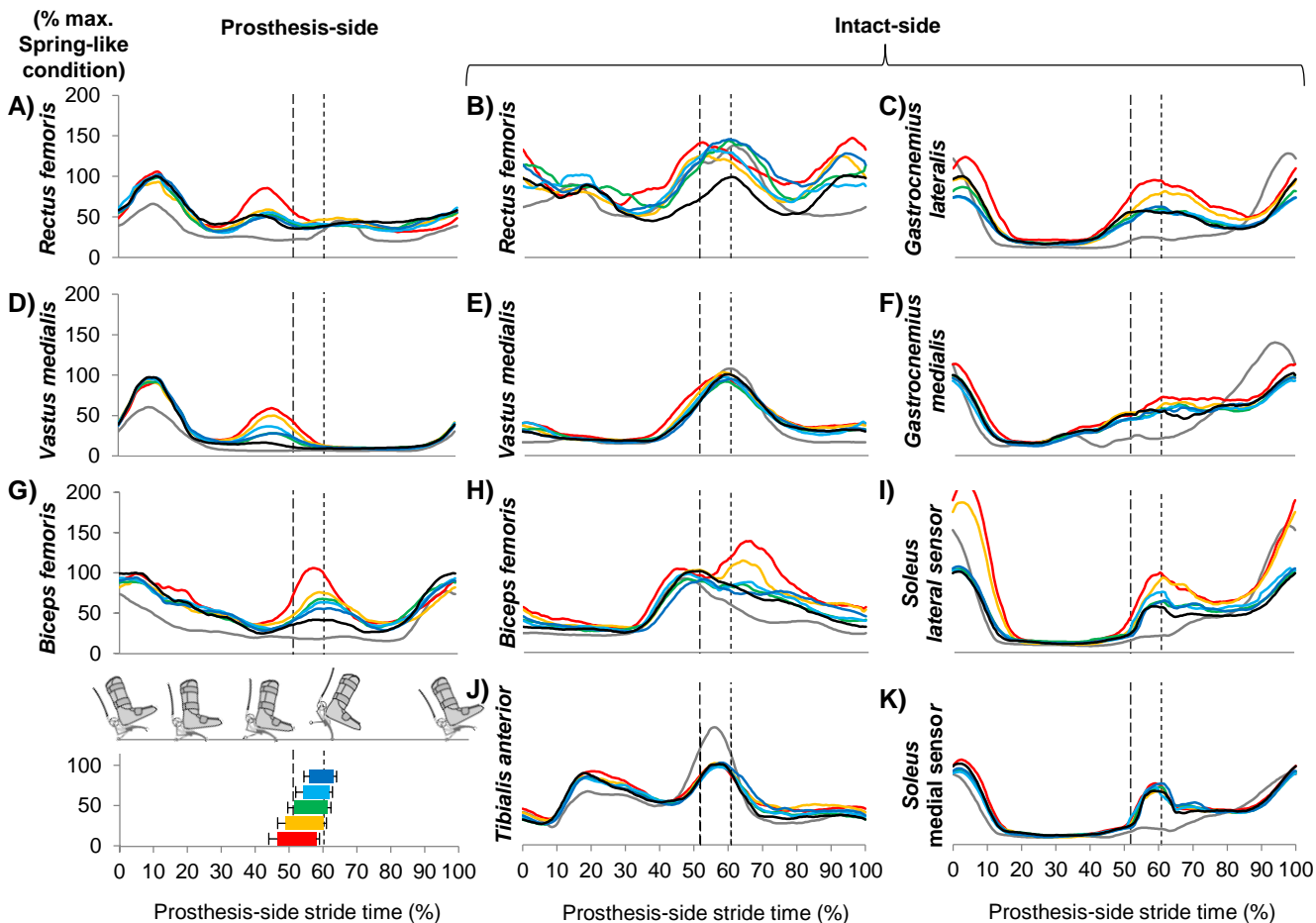

Supplement: Additional file 3: Figure S2. — Electromyography. (A-C) Prosthesis-side rectus femoris, vastus medialis and biceps femoris electromyograms. (D-K) Intact-side rectus femoris, vastus medialis, biceps femoris, tibialis anterior, gastrocnemius lateralis, gastrocnemius medialis, soleus lateralis, and soleus medialis electromyograms. Bar and curve colors correspond to Time-torque onsets. Black line is Spring-like condition. Gray line is Normal Walking. Horizontal bars indicate Time-torque period. Vertical dashed lines indicate mean timing of intact-side heel contact and prosthesis toe off. [file 12984_2015_14_MOESM3_ESM.pdf]

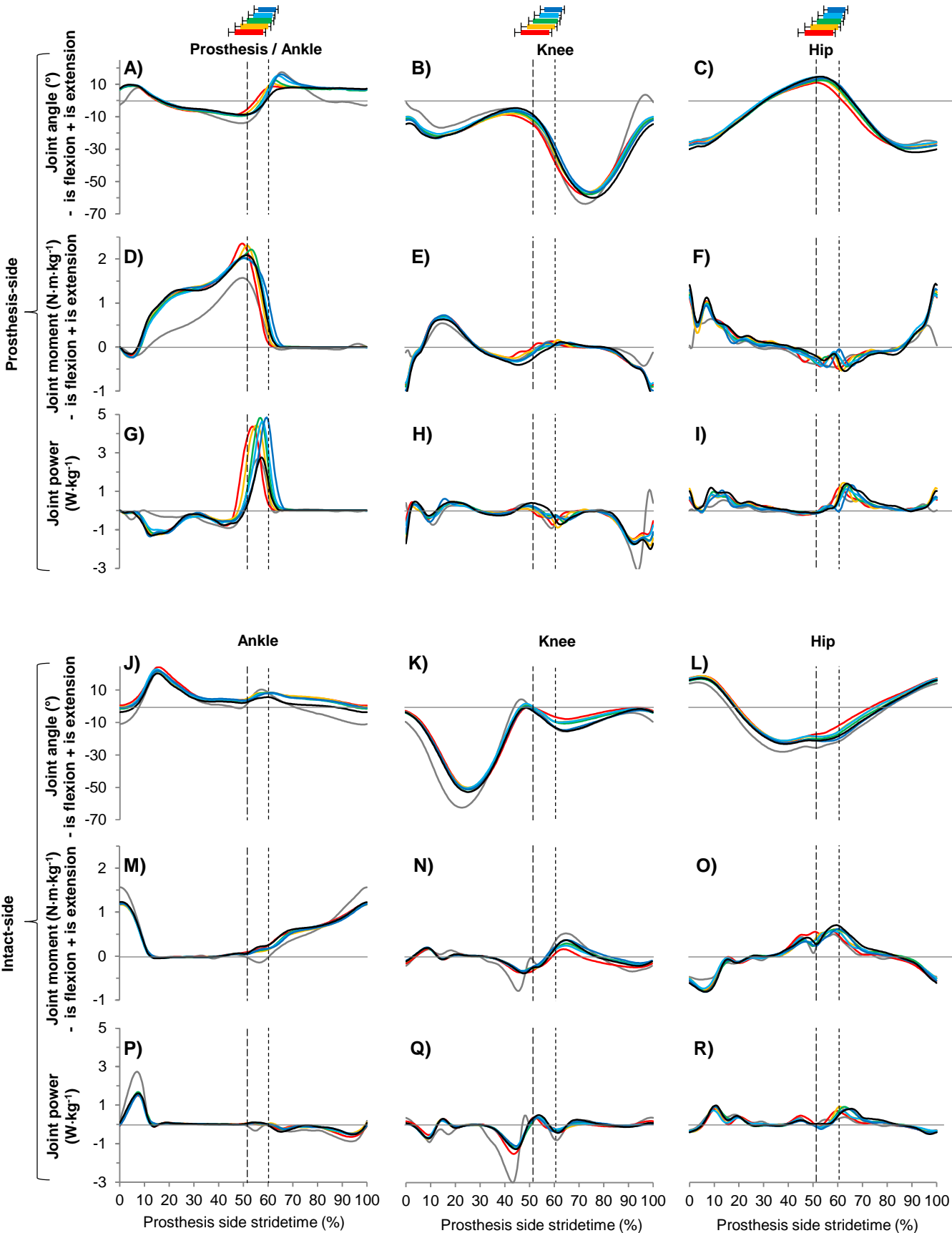

Supplement: Additional file 4: Figure S3. — Sagittal-plane joint kinematics and kinetics. (A to C) Prosthesis-side joint angles. (D to F) Prosthesis-side joint moments. (G to I) Prosthesis-side joint powers. (J to L) Intact-side joint angles. (M to O) Intact-side moments. (P to R) Intact-side joint powers. Bar and curve colors correspond to Time-torque onsets. Black line is Spring-like condition. Gray line is Normal Walking. Horizontal bars indicate Time-torque period. Vertical dashed lines indicate mean timing of intact-side heel contact and prosthesis toe off. [file 12984_2015_14_MOESM4_ESM.pdf]

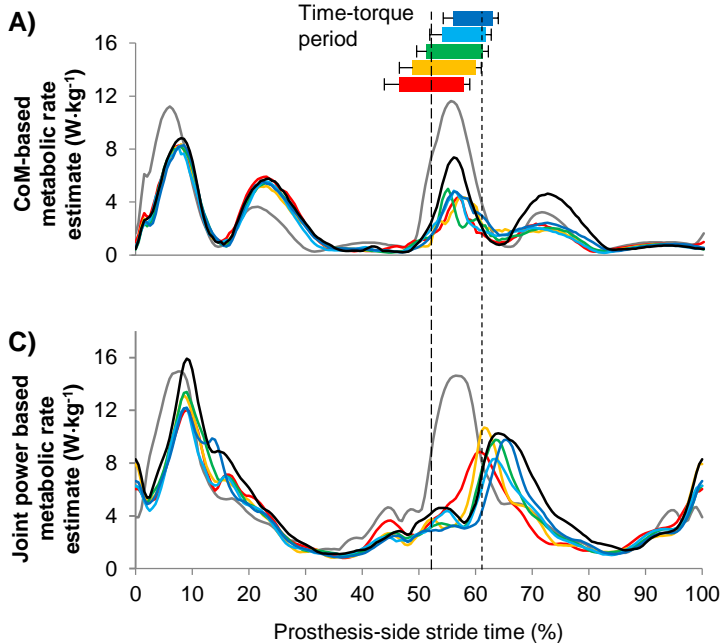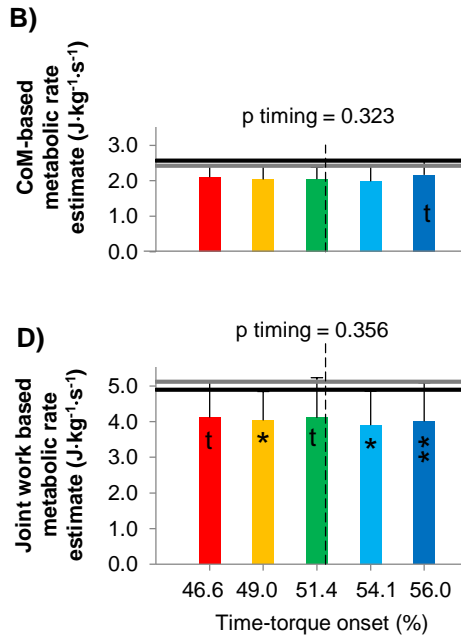

Supplement: Additional file 6: Figure S5. — Metabolic rate estimates. (A) Estimated metabolic rate based on center-of-mass power, which implicitly assumes all work is performed by muscle fascicles. The biological component of prosthesis-side center-of-mass power was obtained by subtracting prosthesis power from prosthesis-side center-of-mass power. Positive and negative power phases were then divided by their theoretical muscle efficiencies (25% and −120%, respectively). The same process was applied to intact-side center-of-mass power, and the two sides were then added together. (B) Estimated average metabolic rate based on center-of-mass power, assuming that all work is performed by muscle fascicles. (C) Estimated metabolic rate based on total joint power, obtained by dividing positive and negative joint power regions by their respective theoretical muscle efficiencies (25% and −120%, respectively) and adding the resulting trajectories for all human joints. (D) Average estimated metabolic rate based on total joint power. Bar and curve colors correspond to Time-torque onsets. Black line is Spring-like condition. Gray line is Normal Walking. Horizontal bars indicate Time-torque periods. Vertical dashed lines indicate mean timing of intact-side heel contact and prosthesis toe-off. Error bars are inter-subject standard deviation. P-values are from repeated measures ANOVA on timing bins. Symbols inside bars indicate significant differences from Spring-like condition. ** = p ≤ 0.01, * = p ≤ 0.05, t = p ≤ 0.1. [file 12984_2015_14_MOESM6_ESM.pdf]
