# Supplementary material for: The influence of push-off timing in a robotic ankle-foot prosthesis on the energetics and mechanics of walking
Source: J Neuroeng Rehabil. 2015 Feb 22;12:21. doi: 10.1186/s12984-015-0014-8 (PMC4404655; doi:10.1186/s12984-015-0014-8)
Supplement: Additional file 5: Figure S4. — Perception scores. Mean perception scores for each Time-torque bin compared to the Spring-like condition. Preference was reported on a scale from −10 to +10, where −10 was ‘cannot walk’ and +10 was ‘walking is effortless’. Error bars are inter-subject standard deviations. P-value is from a repeated measures ANOVA on timing bins. Symbols inside bars represent significant differences versus Spring-like condition. Brackets represent pair-wise differences between conditions. ** = p ≤ 0.01, * = p ≤ 0.05, t = p ≤ 0.1. [file 12984_2015_14_MOESM5_ESM.pdf]

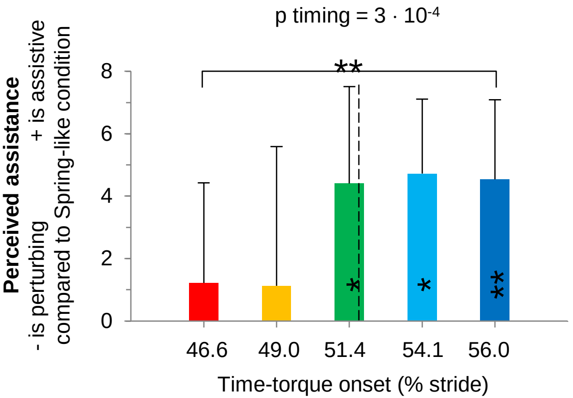

p timing = 3 · 10-4
Perceived assistance
- is perturbing + is assistive
compared to Spring-like condition
Time-torque onset (% stride)
46.6
49.0
51.4
54.1
56.0
0
2
4
6
8
**
*
*
**
